# Heart work: Indigenous doulas responding to challenges of western systems and revitalizing Indigenous birthing care in Canada

**DOI:** 10.1186/s12884-021-04333-z

**Published:** 2022-01-16

**Authors:** Caroline Fidan Tyler Doenmez, Jaime Cidro, Stephanie Sinclair, Ashley Hayward, Larissa Wodtke, Alexandra Nychuk

**Affiliations:** 1 University of Minnesota, Minneapolis, USA; 2 University of Winnipeg, 515 Portage Avenue, Winnipeg, Manitoba R3B 2E9 Canada; 3 University of Manitoba, 66 Chancellors Cir, Winnipeg, MB R3T 2N2 Canada; 4 Kishaadigeh Collaborative Research Centre, University of Manitoba, Winnipeg, Canada; 5 Kishaadigeh Collaborative Research Centre, University of Winnipeg, 515 Portage Avenue, Winnipeg, Manitoba R3B 2E9 Canada

**Keywords:** Indigenous, Doulas, Pregnancy and birthing care, Maternal health, Infant apprehensions, Colonialism, Canada

## Abstract

**Background:**

In Canada, there has been a significant increase in the training of Indigenous doulas, who provide continuous, culturally appropriate support to Indigenous birthing people during pregnancy, birth, and the postpartum period. The purpose of our project was to interview Indigenous doulas across Canada in order to document how they worked through the logistics of providing doula care and to discern their main challenges and innovations.

**Population/setting:**

Our paper analyzes interviews conducted with members of five Indigenous doula collectives across Canada, from the provinces of British Columbia, Manitoba, Ontario, Quebec and Nova Scotia.

**Methods:**

Semi-structured interviews were conducted with members of the five Indigenous doula collectives across Canada in 2020 as part of the project, “She Walks With Me: Supporting Urban Indigenous Expectant Mothers Through Culturally Based Doulas.” Interview transcripts were approved by participants and subsequently coded by the entire research team to identify key themes.

**Results:**

Our paper examines two themes that emerged in interviews: the main challenges Indigenous doulas describe confronting when working within western systems, and how they navigate and overcome these obstacles. Specifically, interview participants described tensions with the biomedical approach to maternal healthcare and conflicts with the practice of Indigenous infant apprehension. In response to these challenges, Indigenous doulas are working to develop Indigenous-specific doula training curricula, engaging in collective problem-solving, and advocating for the reformation of a grant program in order to fund more Indigenous doulas.

**Conclusions:**

Both the biomedical model of maternal healthcare and the crisis of Indigenous infant apprehension renders Canadian hospitals unsafe and uncomfortable spaces for many Indigenous birthing people and their families. Indigenous doulas are continually navigating these challenges and creatively and concertedly working towards the revitalization of Indigenous birthing care. Indigenous doula care is critical to counter systemic, colonial barriers and issues that disproportionately impact Indigenous families, as well as recentering birth as the foundation of Indigenous sovereignty and community health.

## Introduction

In recent years, increasing numbers of Indigenous women across Canada have been undertaking training to become doulas. Indigenous doulas are birth companions who provide continuous physical, mental, emotional, and advocacy support to Indigenous women and birthing people^1^ throughout pregnancy, labour, birth, and the postpartum period, while also offering culturally appropriate care and knowledge. However, unlike midwives and obstetricians (OBs), doulas are not medically trained and do not deliver babies. Many are trained as full-spectrum doulas to offer care across a wide range of reproductive experiences, including miscarriage, abortion, adoption, and stillbirth. It is worth noting that some Indigenous doulas have opted to use the term “birth helper” instead, as “doula” is often not a familiar term, and “helper” speaks to women’s roles as aunties that have traditionally existed in Indigenous communities. One Indigenous doula in Winnipeg defined her role in familial terms: “It’s just being a sister. It’s being kind. It’s caring for someone else. Especially in that very vulnerable intimate moment…what we’re actually doing is just being a good relative; and I wish that our people would change the word ‘doula’ out for ‘being a good relative’” [1]. Indigenous doula and Indigenous Studies scholar Erynne Gilpin (Michif) and scholar Sarah Marie Wiebe similarly explain in a co-authored chapter that doula care honours and enacts relationality: “For Indigenous birth workers, birth is an act of resurgence…Birth is a process binding us to one another in relationship and accountability. Birth is what connects us to our ancestors before us and to our generations to come” [2].

Research shows that doula support provides numerous benefits to women and birthing people, resulting in better birth outcomes [3–7]. Anthropologist Robbie Davis-Floyd summarizes: “Doulas help reduce the amount of pain women experience, help women cope with that pain, alleviate their anxieties, and deal with the emotional issues that often emerge during labor, thereby shortening the length of labor and reducing the number of interventions performed” [8]. The continuous support of a doula is linked to significant reductions in cesarean births and other interventions, as well as contributing to the birthing person’s satisfaction with their birthing experience [9].

In addition to the typical supports provided by mainstream doulas, Indigenous doulas must take account of the specific challenges that Indigenous women and birthing people face in western healthcare systems. These challenges can include medical racism, the threat of infant apprehension by social workers, and the biomedical model of maternal healthcare, which often denigrates Indigenous birthing autonomy and knowledge. As growing numbers of Indigenous doulas are trained across Canada as part of a broader movement to bring birth back to their communities and keep families together, our paper draws on interviews with Indigenous doulas from five different provinces to illustrate their common challenges and innovations. In doing so, we seek to counter the dearth of research on Indigenous doulas [10] and contribute to a growing conversation about the ways in which Indigenous doulas and Indigenous people are reclaiming birth.

## Background: impacts of colonization on Indigenous women’s experiences of birth

Prior to the medicalization of birth, the arrival of an Indigenous baby was a community event that provided community members and family the ability to celebrate, welcome, and support the babies being born into their Nations with culture, language, and connection to place. As Métis scholar Kim Anderson writes: “The teachings tell us that new life was cherished, and at one time pregnant women, infants and toddlers were nurtured and cared for in that spirit. All community members had roles to play in preparing for new life and ensuring that the proper care was given to the pregnant woman and then the newborn.” [11] As life-givers, Indigenous women and birthing people were revered in their roles as caretakers of the new generations, as well as their Nations. Today, however, harmful settler colonial policies have impacted many Indigenous peoples’ contemporary experiences of pregnancy, birth, and parenthood across Canada. Such policies have intentionally targeted Indigenous families as part of the assimilation process. The Indian Residential School system was operated by churches and financed by the Canadian government between the late nineteenth and late twentieth centuries, resulting in the forcible removal of approximately 150,000 Indigenous children between the ages of three and seventeen from their homes to attend the schools. The explicit aim of these schools was to separate Indigenous children from their families and communities, assimilate them into Canadian society, and eliminate their Indigenous identity, as part of a “conscious policy of cultural genocide” [12]. Many children endured illness, exploitation, and abuse at the schools, and thousands died. Devastating disruptions to families caused by the Residential School system resulted in intergenerational impacts for Indigenous people who had been severed from their communities and denied love, knowledge, and guidance from their own parents [13]. These long-lasting effects are also evidenced in the fact that Residential School survivors and their descendants have been documented to suffer from poorer physical and mental health outcomes than those who did not attend the schools [14]. Other colonial efforts that undermined Indigenous families and Nations included the marginalization of Indigenous midwifery and the transfer of medical care from communities to hospitals. During the twentieth century, racially segregated Indian Hospitals were established by the federal government as part of a broader assimilationist agenda [15]. Some of these institutions conducted medical experiments on patients, including a 1933 experimental trial of a tuberculosis vaccine on First Nations infants at the Fort Qu’Appelle Sanatorium; 12 % of these infants died before they turned one [16]. Indigenous communities across Canada have also been significantly impacted by an ongoing mandatory evacuation policy, whereby pregnant First Nations people living on rural and remote reserves are sent to larger towns or cities between 36 and 38 weeks to give birth in hospitals [17]. While these policies were advanced out of purported concern for the health of Indigenous parents and infants, archival research conducted by Gender Studies scholar and midwife Karen Lawford (Lac Seul First Nation) and anthropologist Audrey Giles reveals that such efforts were in fact intended to undermine Indigenous birthing practices and pressure Indigenous people “into accepting the Euro-Canadian biomedical model” [18, 19]. As Gilpin and Wiebe state, “Our teachings, our medicines, our songs and our ways were regarded as alternative, less scientific, and inadequate for the delivery of healthy and safe births” [20]. Michi Saagiig Nishnaabeg scholar Leanne Betasamosake Simpson explains how the transfer of birth from the home to hospitals severed Indigenous people from their systems of support and birthing expertise:

*The birth of a child became something our women had to endure alone, rather than celebrated with the support of her extended family and community. Women were medicated and hospitalized, told that we could not give birth without the assistance of western medicine. White doctors, who were “experts” on birth, replaced our midwives and displaced our confidence in our bodies, our reliance on our traditional knowledge, and our trust in our clans, our spirit-helpers, and our ancestors. Our midwives, aunties and grandmothers were not allowed in delivery rooms, and neither were our medicines, our singing, our drumming, and our birthing knowledge…We were told that for the safety of our babies we needed medical intervention and to rely on the western medical system; to do anything else, we were told, would be irresponsible* [21].

While the western medicalization of birth is typically justified as a measure to improve maternal and infant health outcomes, Indigenous women and birthing people in Canada continue to experience higher rates of adverse birth outcomes than non-Indigenous women, as well as two times the risk of maternal mortality compared to the overall Canadian population [22]. These policies and practices also diminish key aspects of Indigenous peoples’ health, as scholars Karen Lawford, Ivy Bourgeault and Audrey Giles explain: “The maternity care services provided to First Nations women separate them from family and community support, remove them from their land, advance a colonial agenda, and hinder their self-determination” [23]. Furthermore, while the transfer of birth to hospitals is meant to ensure safety, western healthcare spaces are often experienced as unsafe by Indigenous people. The harmful impacts of medical racism against Indigenous people in Canadian healthcare systems are increasingly under scrutiny, as Indigenous people have suffered and even died from what historians Mary Jane Logan McCallum (Munsee Delaware Nation) and Adele Perry term “structures of indifference” in hospitals [24]. A recent report on the issue of medical racism in British Columbia found that Indigenous people encounter widespread systemic discrimination, and that this discrimination has a notable gendered dimension: “This stereotyping, discrimination and prejudice results in a range of negative impacts, harm, and even death. Indigenous women are particularly impacted.” The report adds that Indigenous women testified to “particular feelings of unsafety” [25].

In addition to experiencing the loss of birth in the community, Indigenous women and birthing people across Canada have also been subjected to the reproductive violence of forced or coerced sterilizations [26]. In the 1930s, the provinces of British Columbia and Alberta made sterilization legal. Scholar Karen Stote’s research reveals that many Indigenous people were coercively or forcibly sterilized under the pretext of them being “mentally defective” [27]. And despite the laws being phased out in the early 1970s, a recent proposed class-action lawsuit has found over one hundred reports of Indigenous women and birthing people from different Canadian provinces stating they were forcibly or coercively sterilized in the past few years, with some cases occurring as recently as 2018 [28]. These examples make clear how Indigenous birthing peoples’ bodies and reproductive capabilities have historically been and continue to be key targets of settler colonial regulation and power.

Another stark example of the way in which Indigenous women and birthing people are subjected to reproductive injustice by settler colonial institutions and policies is the crisis of Indigenous infant and child apprehension by child welfare agencies. Indigenous infants are sometimes taken from their mothers almost immediately after birth in a policy known as birth alerts. Indigenous people in Canada experience vastly disproportionate rates of infant and child removal in comparison to non-Indigenous people; 2016 Census data indicates that while Indigenous children ages 0–14 make up only 7.7% of the population of Canada, they account for 52.2% of children in foster care [29]. These removals are linked to adverse health outcomes for both children who have been removed and the mothers/birthing parents whose children are taken from them. One study showed that mothers/birthing parents whose children are apprehended have worse mental health outcomes than those whose children had died [30]. Moreover, mothers/birthing parents whose first child was apprehended have been documented to be at a much higher risk of inadequate or no prenatal care in subsequent pregnancies compared with mothers/birthing parents with no history of involvement with child welfare services [31]. These findings are worth emphasizing because they reveal the ways in which the impact of infant and child removal causes long-lasting detrimental effects on Indigenous parents’ mental and physical health, as well as contributing to cycles of mistrust and fear of healthcare institutions. While child removal is ostensibly enacted to ensure the safety and well-being of children, a recent review of the birth alert system led Manitoba’s former Families Minister Heather Stefanson to state in 2020 that: “what we found is that there’s no evidence to prove that this [birth alert policy]... increases the safety of children in any way” [32]. The 2014 death of 15-year-old Tina Fontaine, who was murdered in Winnipeg while in the care of Manitoba’s Child and Family Services, has catalyzed further scrutiny of child welfare agencies. Fontaine’s death also drew attention to the links between child removal and the crisis of missing and murdered Indigenous women and girls [33]. Indeed, Canada’s National Inquiry into Missing and Murdered Indigenous Women and Girls included Call for Justice #12.8, which recommends an immediate end to the birth alert policy [34]. Soon after the publication of the Inquiry, the provinces of British Columbia and Manitoba officially announced the termination of the policy. Despite this announcement, however, the province of Manitoba stated that “newborns can still face apprehension if a situation is deemed unsafe,” and Indigenous infants are still being taken from their parents [35]. The ongoing crisis of Indigenous child apprehension is a common and devastating feature of Indigenous peoples’ experiences of birth across Canada.

Notwithstanding these efforts to undermine Indigenous families and birthing traditions, many practices and teachings have powerfully persisted in Indigenous communities. In the current moment, with a growing public awareness of the reclamation of Indigenous birth, increasing numbers of Indigenous people are returning to their families and communities to seek out this knowledge. Indigenous midwives and doulas are leading trainings and encouraging more Indigenous people to become involved in their communities as birth helpers. The continuance, adaptation and recovery of these practices signify a concerted effort to reclaim Indigenous birthing roles, relationships and teachings within a biomedical, western healthcare context.

## Methods

Our paper draws on semi-structured interviews conducted in preparation for a larger project, “She Walks with Me: Supporting Urban Indigenous Expectant Mothers Through Culturally Based Doulas,” which is dedicated to the creation of an urban Indigenous doula program in Winnipeg, Manitoba. Our research team decided to conduct interviews with one member from five different Indigenous doula collectives across Canada (British Columbia, Manitoba, Ontario, Quebec and Nova Scotia) in order to learn how they worked through the logistics of providing doula care. We also sought to discern best practices for supporting Indigenous doulas mentally, emotionally, and financially so that they are not over-worked or exploited.

This research received approval from the Human Ethics Review Boards at the University of Winnipeg and the University of Minnesota. The University of Winnipeg Human Research Ethics Board approved the research presented in this paper that was conducted by Cidro, Hayward, Sinclair, Nychuk and Wodtke. The University of Minnesota Institutional Review Board approved the research presented in this paper conducted by Doenmez. Participants were contacted via email and provided verbal consent prior to the interviews, in accordance with the ethics protocols approved by these two review boards. The first interview was conducted in person in the participant’s home city; however, due to the COVID-19 pandemic, the subsequent four interviews were conducted virtually via Zoom. Two to five members of the research team participated in each of the semi-structured interviews.

A key dimension of our research approach was to listen deeply to the participants’ stories, lived experiences and theories of pregnancy, birth, medical racism, healthcare and doula support to focus our analysis on the themes they indicated were most salient. Our results are profoundly impacted by the doulas’ narratives; in this sense, our project viewed stories as “both method and meaning” [36]. Importantly, our research includes stories about the doulas’ own pregnancies and births, as well as insights gleaned from their experiences as doulas supporting other birthing people.

Because we recognize that the stories and information provided to us by our participants are gifts [37], we sent each of our participants an honorarium and a gift to provide a small form of compensation for their valuable time and the knowledge that they shared with us.

Following the interviews, the recordings were transcribed verbatim and returned to participants for review and edits. Each participant was provided at least 3 weeks to review the transcript. Once the transcripts had been approved by participants, the research team developed a coding framework and transcripts were coded both individually and then as a group through the constant comparative method and drawing from grounded theory [38–40]. This intensive process allows for greater reflection among the research team on the themes and examples, with priority given to the participants’ intentions and interpretations. Through the coding process, we identified dominant themes across all interviews. Members of the research team selected different codes to analyze in their respective papers. Drafts of each of these papers were sent to participants for their review to ensure that we were accurately representing their stories and experiences. The following paper analyzes two of the main themes that were discerned through this process: “Navigating western systems” and “Troubleshooting, problem-solving and innovation.”

### Theoretical framework: Indigenous feminisms

We employ an Indigenous feminist framework to analyze the insights provided by the project participants. For the purpose of our analysis, Indigenous feminist theories provide us with several critical, interrelated considerations to advance our understanding of the stakes and impacts of Indigenous doula care. Firstly, Indigenous feminist theories demand that we take account of the gendered and heteropatriarchal nature of settler colonial power and how it impacts Indigenous women’s and birthing peoples’ lives and bodies [41]. While attending to these concerns, Indigenous feminisms also unsettle colonial categories, assumptions, and discourses to reassert Indigenous narratives, theories and ways of being and knowing [42]. In this sense, Indigenous feminisms are “about recognizing, naming, and discarding the worldview forced, reinforced, and enforced by this colonial experiment…and picking up the teachings and practices of our ancestors.” [43] According to Native American Studies scholars Melanie Yazzie (Diné) and Cutcha Risling Baldy (Hupa, Yurok, Karuk), Indigenous feminist approaches are unique in their “commitments to Indigenous life and self-determination in all respects” as well as their emphasis on relationality; they conclude that “to be a good relative is to be an Indigenous feminist” [44]. Similarly, Indigenous education scholar Tasha Spillett (Cree) explains that Indigenous women “are intrinsic to the reconnecting of our families, ending the abduction of our children from the custody of the child welfare system…So that’s the goal of Indigenous feminism: protecting our land and waters, and putting our families back together again” [45]. Critically, Indigenous feminisms center the sovereignty and well-being of Indigenous women, children, and gender-diverse people as core foundations of Indigenous political resurgence and nation-building [46, 47]. Taken together, these Indigenous feminist interventions offer us a theoretical framework that involves critical analyses of colonial systems of power and patriarchy, a re-centering of the self-determination of Indigenous women and non-binary people, an ethics of renewing relationality, and a commitment to protecting Indigenous families*.* We utilize this framework to consider the efforts of Indigenous doulas to reclaim birth as they challenge the norms and practices of western healthcare systems, while revitalizing Indigenous birthing knowledge and care.

## Results and discussion

In this section, we will analyze two of the main themes that emerged in our interviews. The first, “Western systems,” brings to light some of the dominant challenges that Indigenous doulas uniquely face in their work to care for Indigenous birthing people and families. The second, “Troubleshooting, problem-solving and innovation” reveals ways in which Indigenous doulas both navigate the pressures of these systems while also responding attentively to the specific needs of their clients and communities. One participant termed this dedicated practice of relationality and care “heart work.” We offer a brief illustration of the creative, visionary nature of Indigenous doula care, examining how this heart work is profoundly centered on supporting the birthing person and their family, while also interrupting and refusing colonial, racialized and gendered dimensions of western maternal healthcare.

### Western systems

#### Conflicts with biomedicine

Across our interviews, participants describe the challenges of encountering the biomedical approach to maternal health and birth. We use “biomedical” to refer to the dominant model of care in western hospitals wherein pregnancy and birth are seen as pathological conditions to be managed by professionals who are trained within this dominant model of care. In this model, OBs, not birthing people, are assumed to have control over the birth [48–50]. Scholars have noted how this model of birthing care renders birthing people as passive recipients of an OB’s expertise, surveillance and interventions, which can leave birthing people feeling disempowered, invisible and/or disconnected from their own bodies [51–53]. Moreover, they note that such a model of care, rather than being based on scientific evidence, is often in fact reflective of ritualized patriarchal and technocratic values [54]. Indigenous women and birthing people in Canada are subjected not only to these gendered expectations, but also to racialized and colonial values.

Participants in this study describe conflicts with this model of care both in relation to their own personal experiences of giving birth and the births they had attended. One specific area in which this is made clear is the repeated theme in our interviews of birthing people not being able to make their own choices while they are in labour, as well as being pressured into inductions and cesarean births. One participant notes:

*…We see a lot of first-time moms, who have been shamed for weight gain, induced for non-essential reasons. We’ve had moms told, “The baby is definitely going to be too large to even try” and had cesareans, and they ended up having a 6-pound baby. Just so many things were said to people to deter their birth outcomes, and I guess we just wanted to try to level the playing field a little bit, and just try to bring some support. We really believe in the power of women supporting women.*

In this doula’s experience, first-time and younger parents are especially vulnerable to being subjected to judgment and unnecessary interventions. She has observed how doula support can help mitigate against these kinds of interventions and result in more affirming and positive births. Further emphasizing the need for better birthing relationships and experiences, this same participant describes how her own experience of giving birth for the first time was disempowering:

*I had a rough initiation with a male doctor who thought I was overdue, and another male doctor joked about being known for “always getting the girls going,” i.e. helping to start labour. So that kind of comment was made to me in my first pregnancy and it felt very inappropriate…I ended up being taken to the OR for a possible cesarean as baby was said to be in second stage arrest, and there were all sorts of things happening that we had no control over. I just kept asking them if I could get on my hands and my knees, and the response was, “Absolutely not, absolutely not.” I had to just stay on my back with internal fetal monitors. I just knew instinctively that this wasn’t the way my body wanted to birth this baby.*

Ultimately, this participant was pressured into having a cesarean and recounts the memory of this experience as a “fear-based” birth. She was subjected to a distasteful comment from a male OB, as well as being forced to labour on her back against her wishes. Later in our interview, she explains how she sought out a doula for the birth of her second child. The support of her doula resulted in a transformative, overwhelmingly positive experience of pregnancy and delivery, which inspired her to begin working with pregnant people in her community. She remarks: “It was just from that moment on when I saw how different a labour could be, from an epidural in an OR to what I was able to do at the next birth, that I knew I wanted to support people this way.” Here we see how a doula’s own birthing experiences can be foundational for inspiring their involvement in reclaiming birth for other Indigenous people.

Many of the unwanted interventions that Indigenous women and birthing people face in delivery rooms can be linked to the pervasive biomedical concern with managing western concepts of “risk.” In addition to justifying medical interventions, the concern with mediating risky births also underlies the evacuation policy [55]. However, as social anthropology researcher Rachel Olson observed in her ethnographic fieldwork in medical centers in Winnipeg, the narrow focus on managing biological risk in delivering babies does not account for the many other forms of vulnerability or harm that Indigenous birthing people may experience. These can include nonconsensual procedures and violations of their bodies, extreme isolation, alienation, and the risk of infant apprehension [56].

These negative effects, which some Indigenous birthing people face during pregnancy and birth within western healthcare systems, often remain with them for years afterwards. One participant describes how many of the Indigenous people who attend their initiative’s Indigenous doula trainings carry unresolved feelings of sorrow and distress from their experiences of pregnancy and birth:

*And what we’ve found in our first training is that a lot of women have grief and loss around pregnancy. So, we had expected that to be a very short time of the training, or smaller portion of the training, and it turned out to be very extensive, like half a day. And so that was kind of an eye-opener for us, that we just didn’t realize the extent of grief and loss for women around pregnancy…we knew that there were negative experiences around birth, obviously, because we did a community consultation. But then bringing women together like that and giving them the opportunity to talk about their birth experiences, we realized that they still had a lot of trauma around those births. And so, it turned into something a lot more than what we thought it was going to be.*

This comment echoes other participants’ discussions of the need for more training and support to help Indigenous doulas address grief, loss and trauma—both in respect to their own experiences as a birthing person and to better equip them to support other birthing people in their doula work.

Another example of how unequal power dynamics between healthcare professionals and Indigenous birthing people can result in lasting impacts emerged in discussions of informed consent. One participant noted that informed consent is one of the primary concerns that Indigenous doulas in her group typically feel they need to address with OBs. Another participant observes that, in Indigenous doula trainings she had helped to facilitate, attendees have shared many stories of nonconsensual treatment by OBs and nurses:

*And so, when we talk about discrimination and racism from health care providers, and just practices that undermine and diminish people’s consent, like being touched without asking first, and that kind of thing…everybody in the room is nodding and everybody has stories and there’s tears. I think it’s a beautiful thing about our training- I feel like it’s giving Indigenous people and women and child-bearing people specifically, this opportunity to just feel seen.*

It is notable that in this and the prior example, the Indigenous doula trainings themselves became important spaces of heart work for processing and healing from negative medical experiences, as part of the preparation to provide safe care to the people and families they work with. This finding suggests that Indigenous doula trainings are sites of validation and empowerment for the doulas through the sharing of stories. Further, participating in a training builds networks of care, which are made possible through the revitalization of Indigenous birth work and are transformative for the doulas themselves.

Despite the many concerns that we heard about the relationships between healthcare providers and Indigenous people, one participant emphasizes how she has found that certain OBs are in fact quite supportive of Indigenous doulas. She adds that the work of other Indigenous birth workers has helped to begin to shift the culture in the local hospitals where she attends births:

*Some doctors are understanding and see how a doula can benefit a birthing person. I believe a lot of work has already been done for us in the surrounding hospitals. Indigenous practices are already being respected there. Saving the placenta is treated as a given when an Indigenous woman goes in, as well as having multiple family members present in the birthing room and a waiting room full of more family. Having a quiet birthing room is something that’s respected as well. The way has already been paved so that the doctors and nurses in the hospital respect these practices.*

Her quote reveals how the ongoing efforts of Indigenous midwives, doulas and other healthcare workers can help to educate OBs and nurses, leading to improved interactions between them and Indigenous birthing people. These examples highlight how Indigenous doulas’ work can involve countering dominant, biomedical birthing standards to advocate for Indigenous birthing care that centers the birthing person and their knowledges, boundaries, and visions.

Another participant stresses that their Indigenous doula collective’s approach to conceptualizing and delivering care is distinct from western healthcare in that it takes into account the family and community of the birthing person:

*Our model of care that we’re creating together is really a family and community-centered model of care, which is very different from a western patient-centered model. So, when we are providing care, we have to take things into account that are not just impacting individuals but also the larger social context and their health.*

Moreover, the participant mentions that this commitment diverges from the standard approach disseminated in one of the national doula training programs, which fails to address issues such as poverty, housing insecurity, mandatory evacuations, or infant apprehensions. When her Indigenous doula collective first formed, she and the other doulas came together and discussed the kinds of supports they would want to be able to provide to Indigenous community members, grounded in their commitments to decolonization and Indigenous resurgence:

*…to serve Indigenous women who are incarcerated; there’s a big prison about an hour away from here. We also wanted to create partnerships with women who were coming into Vancouver from rural and remote communities, so trying to find ways to connect with communities around the province, to let them know that there are Indigenous support workers who could support them when they came here. And supporting families who might be having their children taken away, or adopted. Just sort of all the kinds of different things that affect Indigenous families and mothers that we wanted to support and connect to in our work.*

Their expansive vision of care reflects how Indigenous doulas are being attentive the many social determinants of health that can inform their clients’ experiences of pregnancy, birth, and parenthood [57]. Far from viewing patients as individualized, anonymous bodies, these Indigenous doulas are seeking to account for the various, intersecting systems and factors that disproportionately impact the people they support.

Additionally, several participants described the ways in which they worked to build trusting and deep connections with the people and families they work with. Their model of birthing support views relationships as a critical aspect of the birth, which stands in stark contrast to what one doula described as “just a transaction, like an OB-led birth can be.” As scholar Gladys Rowe (Fox Lake Cree Nation) has shown in her research on Indigenous doulas, the emphasis on relationships inherent to Indigenous doula care links their birth work to Indigenous resurgence. Rowe’s scholarship underscores the importance of doulas helping to foster modes of care and relationships which seek to “support sovereignty over Indigenous bodies and lands,“ [58] which speaks to Indigenous feminist understandings of decolonization and nation-building as emerging from an ethics of promoting life and relationality.

### Infant and child apprehension

Another example of a western system that many Indigenous doulas are confronting in their work is the crisis of Indigenous infant apprehension and child removal. Although the different doula initiatives we spoke with had varying levels of interaction with child welfare services, multiple participants stated that preventing infant and child removal is a defining feature of Indigenous doula care. Through this commitment to protecting Indigenous families, the doulas are enacting a core feature of Indigenous feminism by working to keep infants with their parents. This speaks to Tasha Spillett’s previously cited definition of the goal of Indigenous feminism involving “putting our families back together again.” Several participants asserted that Indigenous doula support can make the difference between infants and children being apprehended by social workers or staying with their families. For example, one participant tells us:

*…one additional issue is that we have experienced racism effects. We’ve had social workers called in when they thought a mom wasn’t doing what they believe is the “norm.” We’ve fortunately had some fairly good results, as we were there throughout the visits with Department of Community Services, but we know there’s likely many others that are not so fortunate if they do not have supports with them.*

Another participant describes how, for many of the families she works with, “there’s just always this mistrust and fear” when they interact with the healthcare system, due to the constant, underlying threat of child removal. She observes that a large part of her role as a doula is to be present with Indigenous families as a support person and as a witness, and that the advocacy of doulas on behalf of their clients can significantly improve communication with healthcare staff and social workers to deter apprehensions.

Another participant is involved in an effort to deconstruct the very categories that are deployed as justification for Indigenous infant and child removal. As a member of a local committee, she explains that they are currently engaged in discussions examining the specific category of “neglect”:

*I sit on that committee and one of the big discussions that they’re having at that committee level is, “Who defines what neglect is?” And “Who decides?”…A lot of times, the moms we work with wouldn’t consider some of the things that they’re doing “neglect,” but according to Child and Family Services, it is neglect.*

She follows up with another specific example of how Child and Family Services (CFS) imposes views that are inconsistent with Indigenous knowledge: discouraging the practice of infants sleeping in the same beds as their parents, commonly called “co-sleeping.” Many Indigenous people would not consider this practice dangerous to their child, but it is viewed in a negative light by the child welfare agencies:

*And so that’s a discussion that we’re having, and it involves lots of different things, like co-sleeping: CFS frowns on co-sleeping. We know most Indigenous people co-sleep with their children, so our take on that is, “How do you co-sleep safely then? So what’s the knowledge around that and how do you do that safely?” But what happens is that moms are lying about co-sleeping with their children because they’re not allowed to, according to CFS.*

From this example, we see how Indigenous doulas are engaged in critically analyzing and interrogating the categories that are mobilized to surveil Indigenous families and to rationalize the apprehension of their children by social workers. In her work as an administrator of a doula initiative, this participant is clear that in order to build trusting relationships with families and provide the best possible care for them, it is critical to understand their perspectives and apply a nonjudgmental approach to support their parenting.

Our participants, however, noted that there are many challenges to dismantling the cultural and social expectations that contribute to Indigenous people being disproportionately targeted for infant and child removal. One participant observes that within the birth work community in her city, non-Indigenous doulas sometimes report Indigenous families for things they perceive to be problematic. She relays the following example of a post she recently saw on a community birth worker Facebook page, where a doula was considering reporting a family to the Ministry of Children and Family Development (MCFD), which is the provincial child welfare agency:

*And one person posted one day that she was providing care to this family and she was noticing that when she would show up in the mornings, the baby hadn’t had their diaper changed yet…and she was wondering if she should bring it to the attention of the Ministry [of Children and Family Development]. And a whole bunch of people, including non-Indigenous people, were like, “No, there’s no reason to do that, it’s not weird at all for people to have not gotten around to the morning diaper change, and usually the morning diaper is quite full!” But that’s so scary! Because people, I think that they have this saviour complex and all these layers of power and privilege that they haven’t checked.*

Importantly, this participant assesses the impulse to report Indigenous parents as part of a saviour mentality, indicating how the rationale of purportedly “helping” Indigenous infants and children often underlies the apprehension of Indigenous children. This same justification has been used throughout generations by colonial actors and institutions to remove Indigenous children from their families and communities, including the Residential School system. This doula explicitly links the tendency to monitor Indigenous parents and seize Indigenous children to a paternalistic colonial mentality. She explains:

*I think it comes from this sense of entitlement over our bodies and our lands. And this idea that they [non-Indigenous people] know better than we do, I mean that’s the epitome of colonization. People are so… it’s so deeply ingrained into them, they don’t, they wouldn’t ever outright admit that that’s what’s going on, but that’s what’s going on.*

Her commentary testifies to how colonial ideologies regarding the inadequacy of Indigenous parents are accompanied by a disavowed sense of righteous superiority that mobilizes and feeds into the crisis-level rates of Indigenous infant apprehensions. In this sense, her comments resonate with the insights of Indigenous feminists who conceptualize Indigenous women’s and children’s bodies as political orders that threaten heteropatriarchal settler power and thus are continually targeted for assimilation by Canadian institutions [59, 60].

The same doula explains how the ubiquitous threat of infant and child removal deters Indigenous people from interacting with western systems and institutions, even when they need certain kinds of support. While reflecting on a personal conflict she experienced with her partner during her own pregnancy, as well as the experiences of the families she has supported in her role as a doula, she notes the dilemma that Indigenous people face:

*I think it’s really difficult because from what I’ve seen from my own personal experience and some of the families that I work with, is that one of the most insidious, tragic parts of colonization, and what it’s impacted, is the infrastructure that we have in communities around family and justice…. when that [interpersonal conflict between couples or families] happens, where do we go, as Indigenous parents, for support when that happens? Because if we turn to the justice system, we often get turned over to the Ministry. If we go into the justice system, the justice system doesn’t heal families, the justice system perpetuates more trauma onto families. And so, where do you go, where is a place you can go that actually won’t just perpetuate more pain for your family and the child? And pass that onto the child? And for so many Indigenous people, like, where do we go?*

Her story illustrates how the child welfare system is entangled with other western institutions and systems, which often discourages Indigenous people from engaging with them out of fear of being reported as inadequate parents. Her comments indicate how the justice system in Canada is experienced as threatening and punitive by many Indigenous people. While this particular doula and her partner were able to work through their issues with the guidance of Elders and ceremony, she is aware that many people do not have access to those kinds of community-based supports. As a result, in the doula trainings that she helps to facilitate, she encourages the doulas-in-training to reflect on what kinds of projects and practices they could cultivate in their own communities to provide safe, Indigenous-led options for families who would benefit from supports that are committed to family and community cohesiveness.

#### Troubleshooting, problem-solving, and innovation

Closely related to the challenges of navigating the western systems of biomedicine and infant apprehensions discussed above, the second key theme that emerged in our interviews was “troubleshooting, problem-solving, and innovation.” This theme encompasses a wide array of examples that draw attention to the intensely creative, adaptive, and responsive nature of Indigenous doula care. In forming their own collectives and administering services, our participants had worked through various challenges to create models of doula care that reflect specific Indigenous values and concerns. This theme primarily emerged through discussions and examples of three subthemes: 1) the development of Indigenous-specific doula training curricula, 2) gatherings to bond and problem-solve, and 3) advocacy to reform a grant program to be accessible to Indigenous doulas.

### Indigenous-specific doula training curricula

Our interviews revealed that one of the ways in which Indigenous doula programs are engaged in inventive and groundbreaking work is through their commitment to creating and frequently updating their own Indigenous doula training curricula. While several doula training programs are offered throughout Canada, they rarely include content by and for Indigenous people. Several participants we spoke with noted that these generic training models often do not equip them to support their clients’ specific needs. In developing their own respective curricula, community input is critical, as one participant explains:

*In the development of the curriculum we have a number of community consultations. And so we met with…practicing Indigenous doulas, we met with Indigenous midwives and other medical professionals that were in the birth field. And we also met with grandmothers, we met with families, so we met with moms who were currently pregnant, and then moms who had also given birth, and then we also met with dads. And I think those were all the groups that we met with. And so we took their experiences, and with the moms, took their experiences and try to address that in our development of the curriculum, and then as well as the dads and to try to include in the training stuff around working with dads and how we can have them more involved in the pregnancy and the whole birthing process. And so then we wrote the curriculum and then we took it back to the grandmothers that we had worked with for validation, and all through it actually, so the grandmothers that we had worked with...those were the women who validated our curriculum.*

This participant’s comment highlights how, rather than looking to western standards, their curriculum reflects the needs and wisdom of their community members, clients, and Elders, and in doing so, enshrines their knowledge, experience, and authority. Two other participants we interviewed also recently created their own curricula to administer Indigenous doula trainings in their respective communities, indicating a growing interest in developing Indigenous-specific trainings to prepare Indigenous doulas to support families. This development indicates that the doula initiatives are involved in reclaiming and disseminating Indigenous knowledges, practices, and roles around pregnancy and birth. These efforts to re-write the doula training curricula exemplify what Native American Studies scholar Cutcha Risling Baldy identifies as the power of Indigenous feminist approaches “as tools for (re)writing, (re)righting, and (re)riteing” [61]. Through their work to re-create the foundational knowledges, tenets, and practices of doula trainings by centering local Indigenous perspectives, Indigenous doulas are unsettling colonial expectations of care, reasserting Indigenous ways of knowing about birth, and honouring the expertise and self-determination of Indigenous women and birthing people.

### Gatherings to bond and problem-solve

Because many of the Indigenous doulas working at these collectives are relatively new to birth work, having a space and process to problem-solve with other Indigenous doulas is essential. The comments we heard suggested that becoming a doula is a long-term learning journey that is maintained by developing trusting relationships between the different doulas in their respective collectives. One participant tells us:

*Our collective structure, much like the structure that we follow with our clients, is very focused on birth stories. When we do have collective meetings, a big component of that is sharing birth stories so we can work through what happened, discuss any triggers they may have felt and then, we can help each other out that way.*

The participant explains that their meetings have become a way for doulas to not only discuss what has happened to their clients, but also to process any of their own trauma that has been triggered through an experience. Another participant describes how her group works through challenging and unique circumstances by coming together and relying on a strong network of community resources:

*Things come up all the time and the way that we solve them is always by talking to each other. And if we don’t know, we have community programs that might deal with some of these issues….We’ve got plenty of programs that deal with so many issues, so if it’s something that we're not strong in, we always go to people who are knowledgeable. And I think that, being that many of us are young moms in the community, it’s easy for us to have access to those resources already…we know who to talk to or where to send clients.*

One participant points out that her group’s discussions became so time-intensive that they decided to narrow down the focus of these sessions:

* We have a staff meeting every Monday morning, so it’s a debrief session. They used to go through every single client and update on every single client and everything that they were doing, which took hours and hours and hours, so now we’ve narrowed it down more to like, “Are you having any challenges? Do you have any, some successes that you want to share with us about your moms? And then anything that you want to problem solve with the other staff?” *

This participant notes that while they had to work to keep the meetings from becoming too long, the doulas in the group are constantly checking in with each other and sharing ideas in group texts. Another participant recounts how organizing a retreat to reconnect, reflect, and brainstorm was a crucial way for her collective to touch base after falling out of touch at one point. This gathering was a way for them to affirm their shared values and acknowledge certain challenges that were emerging around their availability and capacity. Subsequently, the collective decided to shift some of their expectations related to work and communication, and left with a renewed sense of connection, support and a common vision of the care they wanted to deliver to their community. These different examples demonstrate how different doula collectives find meetings to be a critical space to share stories, brainstorm, and problem-solve, while also reinforcing and strengthening the relationships between doulas.

### Reforming funding programs to include Indigenous doulas

Another key example of Indigenous doulas’ innovative problem-solving efforts relates to the issue of funding. Indigenous doulas in many locations across Canada struggle with being adequately compensated for their work. Different collectives rely on distinct payment models, but several participants describe the ongoing challenges of seeking and maintaining sustainable funding sources. To illustrate these challenges, we discuss the following example in depth. In one province, a grant exists to pay doulas who support Indigenous families; however, this grant has previously only been accessible to doulas trained with specific certifications, which excludes many Indigenous doulas who undertook different trainings. Despite Indigenous families wanting Indigenous doulas, many families have only been able to access non-Indigenous doulas because of the funding requirements. One participant learned of this issue first-hand when she became pregnant herself and sought out the support of Indigenous doulas several years ago:

*So that was one of the things that from the beginning our collective wanted to address, was advocacy around this grant program to try to make it more accessible. And the reason why we had found out it had these barriers to access was actually because I, when I was pregnant, I tried to access the grant to pay my doulas, and they were turned down. And so then, as just a client of the grant program, or a recipient of care, I had sent a letter [to the funding organization], saying why I thought that was really problematic- these are Indigenous doulas, I specifically chose them as an Indigenous person for that reason. They are offering me all of these things non-Indigenous doulas cannot offer. And they were really sympathetic, but they were kind of like “Well, our hands are tied, this money, you know these stipulations come from the funder.” And so we ended up finding out that that stipulation came from this concern from the Ministry of Health around medical liability…so there’s always been this major barrier for Indigenous doulas to access this grant, and we thought, “This is such a missed opportunity and it seems like such a simple fix, because the grant program is great, it’s supporting Indigenous families to get doula care, but it could also be supporting the rebuilding of infrastructure in Indigenous communities around birth.”*

Motivated by their vision of bringing birth back, the participant and others in her initiative have strenuously advocated for years for changes in the requirements so that Indigenous doulas can be eligible for funding via this particular grant. Their work resulted in successful changes to the program, which were recently implemented in their province in March 2020. The participant reflects on the significance of their problem-solving approach: “I feel like we’ve really done something that is going to be meaningful to Indigenous people around the province and actually put money in Indigenous peoples’ pockets, and that is like, *yes, we did that.* I’m proud of us.” Because many families cannot afford to pay doulas themselves, Indigenous doulas without access to the grant funding often provide their services for little or no compensation, a dilemma that exists across the country. It is from this example that it becomes apparent Indigenous doulas have to concertedly work to overcome certification and funding challenges to ensure that more Indigenous doulas have access to funding and are able to work in their communities. This doula emphasizes that their work to reform the grant model was part of a bigger project to redefine healthcare and reclaim birth: “And ultimately, long term vision: if we want to improve maternal health outcomes in Indigenous communities, we need to have care in community. And we need to have our own people able to provide this kind of care.”

## Conclusion

In the examples discussed in this paper, we identified several challenges that Indigenous doulas are contending with. It is evident that our participants, despite location of service, face common barriers when navigating the biomedical model of maternal healthcare and the threat of infant and child apprehensions while supporting Indigenous women and birthing people. Moreover, the Indigenous doulas’ stories also attest to the importance of innovative approaches to providing care. These approaches are critical for designing and adapting the curricula of Indigenous doula trainings, working through birth stories and issues together in gatherings, and reforming grant programs to ensure more Indigenous doulas are compensated for their work. The level of creativity and problem-solving required for these Indigenous doulas to meet their clients’ needs directly reflects the fact that western healthcare systems were designed by and for non-Indigenous people. Therefore, we contend that Indigenous doulas are working towards the decolonization of maternal healthcare systems by practicing Indigenous feminist commitments and ethics, in order to support the sovereignty of Indigenous birthing people and their families.

The challenges and innovations discussed in our paper suggest that Indigenous doula care enacts Indigenous feminist concepts in several ways. While a biomedical and colonial perspective situates western healthcare providers as the ultimate authorities and experts on birth and views Indigenous women from a deficit model, Indigenous feminist approaches instead posit Indigenous women’s bodies as sites of self-determination [62]. Indigenous doulas’ heart work exemplifies this concept by countering dominant biomedical birthing norms, putting relationships at the center of their work, and taking account of the broader contexts of their clients’ lives and health. They advocate for birthing care that reflects Indigenous knowledges and values through the development of their own curricula and their efforts to support more Indigenous doulas in their communities. Critically, in their shared commitments to keeping Indigenous families together and preventing newborn apprehensions, Indigenous doulas are involved in the urgent work of challenging the inter-generational colonial violence of infant and child removal. Through this heart work, they are enacting an Indigenous feminist praxis that seeks to bring birth back as the foundation of Indigenous sovereignty and community health.

## Data Availability

Following the consent process, the individual qualitative interview transcripts will not be made publicly available.

## Abbreviations

OR: Operating Room
OB: Obstetrician
CFS: Child and Family Services, the child protection service of Manitoba
MCFD: Ministry of Child and Family Development, the child protection service of British Columbia

## Footnotes

1 In our paper, we use the terms “birthing people” and “birthing parent” to refer to people who give birth but do not identify as women.

## References

[1] Cidro J, Doenmez C, Phanvoulong A, Fontaine A (2018). Being a good relative: indigenous doulas reclaiming cultural knowledge to improve health and birth outcomes in Manitoba, Canada. Front Women’s Health.

[2] Gilpin EM, Wiebe SM, Einion A, Rinaldi J (2018). Embodied governance: community health, indigenous self- determination, and birth practices. Bearing the weight of the world: exploring maternal embodiment.

[3] Koumouitzes-Douvia J, Carr CA (2006). Women’s perceptions of their doula support. J Perinat Educ.

[4] Gruber KJ, Cupito SH, Dobson CF (2013). Impact of doulas on healthy birth outcomes. J Perinat Educ.

[5] Hanley GE, Lee L (2017). An economic model of professional doula support in labour in British Columbia, Canada. J Midwifery Womens Health.

[6] Hodnett ED, Gates S, Hofmeyr GJ, Sakala C (2013). Continuous support for women during childbirth. Cochrane Database Syst Rev.

[7] Dekker R. Evidence on: doulas. Evidence based birth. Originally published March 27, 2013, updated May 4, 2019. Available from: https://evidencebasedbirth.com/the-evidence-for-doulas/. Accessed 23 May 2021.

[8] Davis-Floyd R (2018). Ways of knowing about birth: mothers, midwives, medicine & birth activism.

[9] Bohren MA, Hofmeyr GJ, Sakala C, Fukuwaza RK, Cuthbert A (2017). Continuous support for women during childbirth (review). Cochrane Database Syst Rev.

[10] Ireland S, Montgomery-Andersen R, Geraghty S (2019). Indigenous doulas: a literature review exploring their role and practice in western maternity care. Midwifery..

[11] Anderson K (2011). Life stages and native women: memory, teachings, and story medicine.

[12] What we have learned: principles of truth and reconciliation. The truth and reconciliation commission of Canada 2015: 25. Available from: https://ehprnh2mwo3.exactdn.com/wp-content/uploads/2021/01/Principles_English_Web.pdf. Accessed 11 Oct 2021.

[13] Fontaine L, Forbes L, McNab W, Murdock L, Stout R, Lavell-Harvard DM, Anderson K (2014). Nimâmâsak: the legacy of first nations women honouring mothers and motherhood. Mothers of nations: indigenous mothering as global resistance, reclaiming and recovery.

[14] Wilk P, Maltby A, Cooke M. Residential schools and the effects on indigenous health and well-being in Canada- a scoping review. Public Health Rev. 2017;38(8) Available from: 10.1186/s40985-017-0055-6. 10.1186/s40985-017-0055-6 PMC5809999 29450080

[15] Lux MK (2016). Separate beds: a history of Indian hospitals in Canada, 1920s–1980s.

[16] Lux MK (2010). Care for the “racially careless”: Indian hospitals in the Canadian west, 1920-1950s. Can Hist Rev.

[17] Lawford KM, Giles AR, Bourgeault IL (2018). Canada’s evacuation policy for pregnant first nations women: resignation, resilience, and resistance. Women Birth.

[18] Lawford KM, Giles AR (2012). Marginalization and coercion: Canada’s evacuation policy for pregnant first nations women who live on reserves in rural and remote areas. Pimatisiwin: J Aboriginal Indig Community Health.

[19] Lawford KM (2016). Locating invisible policies: Health Canada’s evacuation policy as a case study. Atlantis: Crit Stud Gender, Cult Soc Justice.

[20] Gilpin EM, Wiebe SM, Einion A, Rinaldi J (2018). Embodied governance: community health, indigenous self- determination, and birth practices. Bearing the weight of the world: exploring maternal embodiment.

[21] Simpson LB, Lavell-Harvard DM, Lavell JC (2006). Birthing an indigenous resurgence. “Until our hearts are on the ground”: Aboriginal mothering, oppression, resistance and rebirth.

[22] Kolahdooz F, Launier K, Nader F, Yi KJ, Baker P, McHugh T-L, Vallianatos H, Sharma S (2016). Canadian indigenous women’s perspectives of maternal health and health care services: a systemic review. Divers Equal Health Care.

[23] Lawford K, Bourgeault IL, Giles AR (2019). “This policy sucks and It’s stupid:” mapping maternity care for first nations women on reserves in Manitoba, Canada. Health Care Women Int.

[24] McCallum MJL, Perry A (2018). Structures of indifference: an indigenous life and death in a Canadian City.

[25] Turpel-Lafond ME (2020). In plain sight: addressing indigenous-specific racism and discrimination in B.C. health care. Addressing racism review, British Columbia ministry of health.

[26] Stote K (2015). An act of genocide: colonization and the sterilization of aboriginal women.

[27] Stote K (2012). The coercive sterilization of Aboriginal women in Canada. Am Indian Cult Res J.

[28] Zingel A (2019). Indigenous women come forward with accounts of forced sterilization, says lawyer.

[29] Backgrounder: Child & Family Services (2018). Indigenous services Canada, Government of Canada.

[30] Kenny K (2018). Mental health harm to mothers when a child is taken by child protective services: health equity considerations. Can J Psychiatry.

[31] Wall-Wieler E, Kenny K, Lee J, Thiessen K, Morris M, Roos L (2019). Prenatal care among mothers involved with child protection services in Manitoba: a retrospective cohort study. Can Med Assoc J.

[32] Funk C, Brohn E (2020). Review finds no evidence birth alerts improve child safety, Manitoba families minister says.

[33] Taylor J (2018). “Tina Fontaine is that direct link” between MMIWG, child welfare system, advocate says at inquiry hearing.

[34] Calls for Justice (2019). Reclaiming power and place: the final report of the National Inquiry into missing and murdered indigenous women and girls.

[35] Laychuk R. Brandon couple reunited with newborn son who was apprehended 2 days after birth. Winnipeg: CBC News Manitoba; 2021. Available from: https://www.cbc.ca/news/canada/manitoba/brandon-family-child-returned-after-hospital-apprehension-1.5899937

[36] Kovach M (2009). Indigenous methodologies: characteristics, conversations, and contexts.

[37] Lavallée L (2009). Practical application of an indigenous research framework and indigenous research methods: sharing circles and Anishnaabe symbol-based reflection. Int J Qual Methods.

[38] Lincoln YS, Guba EG (1985). Naturalistic inquiry.

[39] Charmaz K, Morse JM, Noerager Stern P, Corbin J, Bowers B, Charmaz K, Clarke AE (2021). The genesis, grounds, and growth of constructivist grounded theory. Developing grounded theory: the second generation revisited.

[40] Olson JD, McAllister C, Grinnell LD, Gehrke Walters K, Appunn F (2016). Applying constant comparative method with multiple investigators and inter-coder reliability. Qual Rep.

[41] Arvin M, Tuck E, Morrill A (2013). Decolonizing feminism: challenging connections between settler colonialism and heteropatriarchy. Fem Form.

[42] Goeman M. (re)mapping indigenous presence on the land in native Women’s literature. Am Q. 2008;60(2):295–302.

[43] Gearon J (2021). Indigenous Feminism Is Our Culture. Stanford social innovation review.

[44] Yazzie M, Baldy CR (2018). Introduction: indigenous peoples and the politics of water. Decolonization: Indig Educ Soc.

[45] Monkman L (2017). Indigenous feminism: what is it, and what does the future hold?. CBC Indigenous.

[46] Anderson K, Suzack C, Huhndorf SM, Perreault J, Barman J (2010). Affirmations of an indigenous feminist. Indigenous women and feminism: politics, activism, culture.

[47] Simpson LB, Anderson K, Campbell M, Belcourt C (2018). Centring resurgence: taking on colonial gender violence in indigenous nation building. Keetsahnak: Our Missing and Murdered Indigenous Sisters.

[48] Davis-Floyd R (1994). The technocratic body: American childbirth as cultural expression. Soc Sci Med.

[49] Neiterman E (2013). Sharing bodies: the impact of the biomedical model of pregnancy on Women’s embodied experiences of the transition to motherhood. Health Policy.

[50] Cidro J, Hayward A, Bach R, Sinclair S. Walking with our Most sacred: indigenous birth workers clearing the path for returning birthing to indigenous communities. In: Greenwood M, Stout R, Larstone R, de Leeuw S, editors. Introduction to first nations, Inuit, and Métis determinants of health. Toronto: Canadian Scholars Press. In Press, expected publication 2022.

[51] Martin E (1987). The woman in the body: a cultural analysis of reproduction.

[52] De Vries R (2017). Obstetric ethics and the invisible mother. Narrat Inq Bioethics.

[53] Newnham E, McKellar L, Pincombe J (2018). Towards the humanisation of birth: a study of epidural analgesia and hospital birth culture.

[54] Davis-Floyd R (2018). The rituals of hospital birth: enacting and transmitting the technocratic model. Ways of knowing about birth: mothers, midwives, medicine & birth activism.

[55] Finestone E, Stirbys C, Neufeld HT, Cidro J (2017). Indigenous birth: reconciliation and reproductive justice in the settler state. Indigenous experiences of pregnancy and birth.

[56] Olson R, Neufeld HT, Cidro J (2017). Bearing witness: rural indigenous Women’s experiences of childbirth in an urban hospital. Indigenous experiences of pregnancy and birth.

[57] Leason J (2017). Exploring the complex context of Indigenous women’s maternity experiences in the Okanagan valley, British Columbia by expanding on Aboriginal women’s responses to the Canadian maternity experiences survey.

[58] Rowe GL (2020). Resurgence of indigenous nationhood: centering the stories of indigenous full spectrum doulas.

[59] Simpson A. The state is a man: Theresa Spence, Loretta Saunders and the gender of settler sovereignty. Theory & Event. 2016;19(4):9.

[60] Simpson LB (2017). As we have always done: indigenous freedom through radical resistance.

[61] Baldy CR (2018). We are dancing for you: native feminisms and the revitalization of women’s coming-of-age ceremonies.

[62] Gilpin EM, Wiebe SM, Einion A, Rinaldi J (2018). Embodied governance: community health, indigenous self- determination, and birth practices. Bearing the weight of the world: exploring maternal embodiment.

